# Supplementary material for: Nestin regulates cellular redox homeostasis in lung cancer through the Keap1–Nrf2 feedback loop
Source: Nat Commun. 2019 Nov 6;10:5043. doi: 10.1038/s41467-019-12925-9 (PMC6834667; doi:10.1038/s41467-019-12925-9)
Supplement: Supplementary file 1 — Supplementary Information [file 41467_2019_12925_MOESM1_ESM.pdf]

Supplementary Information

Nestin regulates cellular redox homeostasis in lung cancer through the Keap1-Nrf2 feedback loop

Wang et al.

Supplementary Figure 1

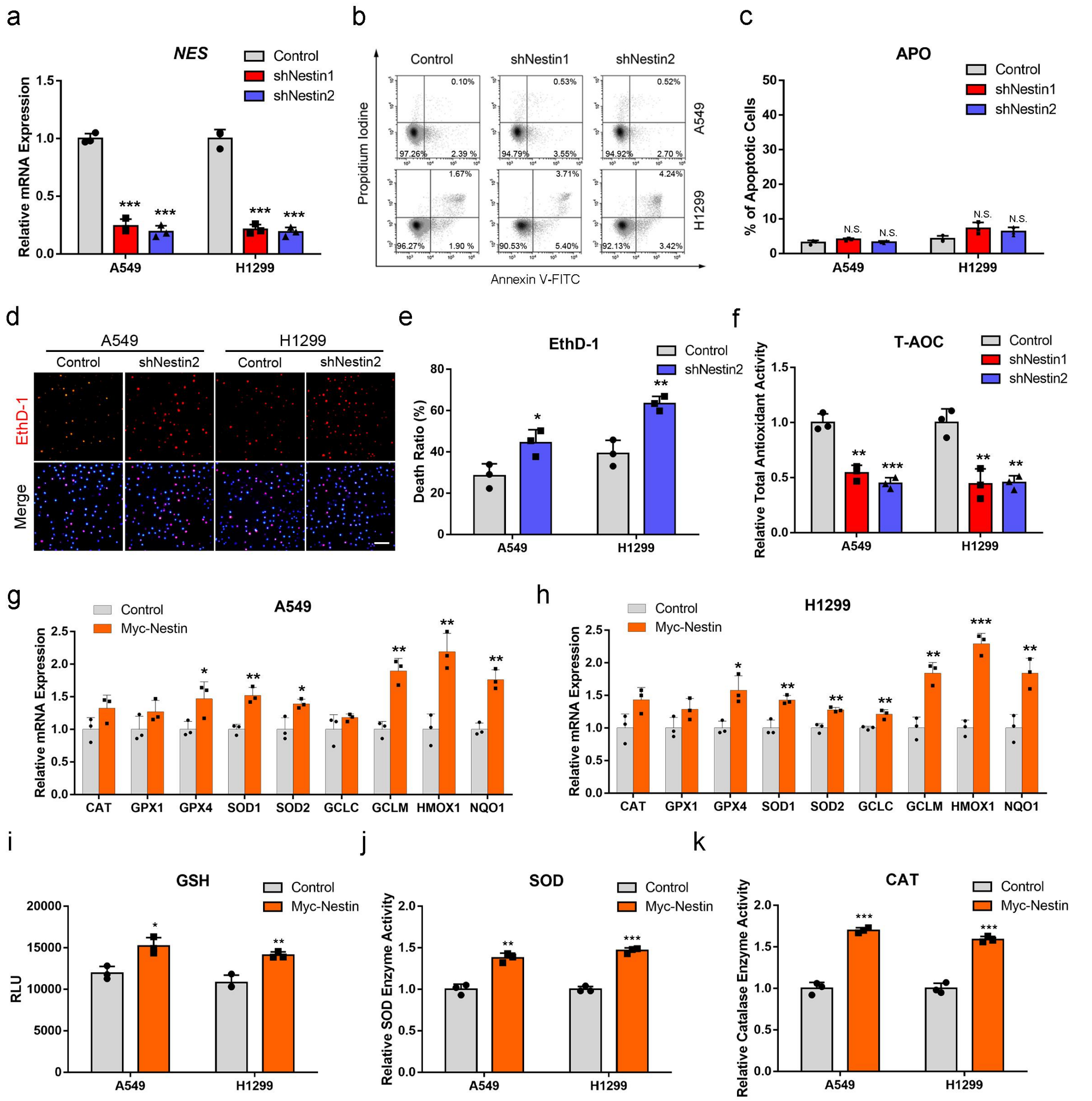

Supplementary Figure 1, relative to Figure 1. Nestin affects antioxidant system in NSCLC cells.

(a) qPCR analysis of the interference efficiencies of the shNestin1 and shNestin2 vectors.

(b) NSCLC cells were transfected with Control, shNestin1 or shNestin2 plasmids. Flow cytometric analysis with Annexin V-FITC and PI was used to detect apoptosis.

(c) Statistical analysis of the total apoptosis rate in NSCLC cells.

(d) NSCLC cells were transfected with Control or shNestin2 plasmids and treated with 200

μM H_2O_2 for 6 h. The LIVE/DEAD viability/cytotoxicity assay was used to ascertain the H_2O_2-induced cell death in NSCLC cells. Scale bar: 50 μm.

(e) Statistical analysis of the death rates of the NSCLC cells described in (d).

(f) NSCLC cells were transfected with Control, shNestin1 or shNestin2 plasmids, and the total antioxidant activity (T-AOC) was assessed.

(g and h) qPCR analysis showing that overexpression of Myc-Nestin in A549 and H1299 cells affects the expression levels of several antioxidation-related genes compared to those seen in control cells.

(i) Analysis of GSH levels in NSCLC cells.

(j) SOD activity in NSCLC cells was examined with a SOD assay kit.

(k) Analysis of CAT levels in NSCLC cells with or without Myc-Nestin.

Data are presented as the means ± SD of three independent experiments. * $P < 0.05$, **$P < 0.01$ and ***$P < 0.001$, Student's *t* test. Source data are provided as a Source Data file.

Supplementary Figure 2

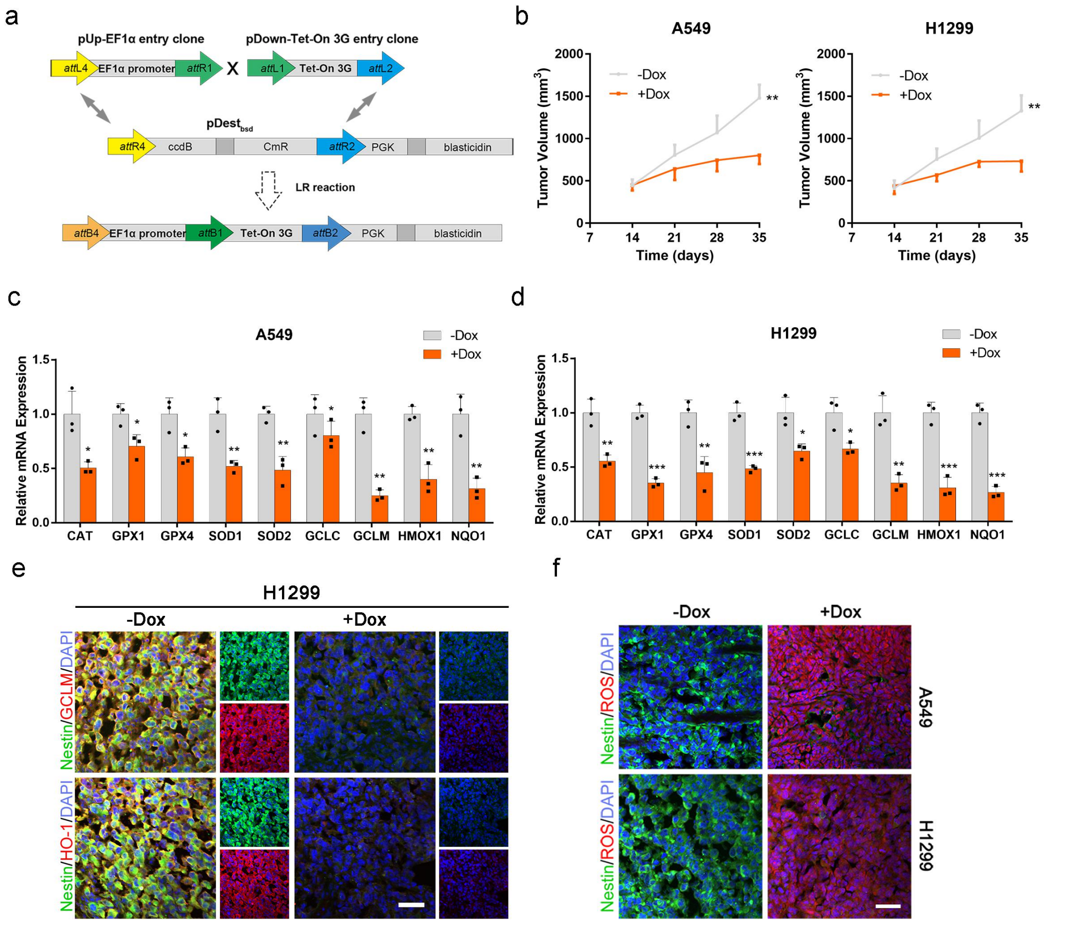

Supplementary Figure 2, relative to Figure 2. Deletion of Nestin in NSCLC cells leads to the attenuation of antioxidant system.

(a) Schematic depiction of how the EF1α-Tet-On 3G-bsd plasmid was constructed via Gateway technology.

(b) The volume of human xenograft tumors in mice treated with Dox shrink compared to the ones without treated with Dox (n=3 per group).

(c and d) qPCR analysis showing that knockdown of Nestin in NSCLC cells via the Tet-On 3G doxycycline-inducible gene expression system reduced the expression levels of several antioxidation-related genes.

(e) Immunofluorescence was used to detect the expression of Nestin, GCLM and HO-1 in human xenograft tumors of mice treated with/without Dox. H1299 cells were labeled with anti-GCLM/HO-1 (red), anti-Nestin (green) and DAPI (blue). Scale bar: 50 μm.

(f) Immunofluorescence was used to detect ROS levels in human xenograft tumors of mice treated with/without Dox. NSCLC cells were labeled with DHE (red), anti-Nestin (green) and DAPI (blue). Scale bar: 50 μm.

Error bars represent the SD of data obtained from three independent experiments. * $P < 0.05$, ** $P < 0.01$ and *** $P < 0.001$, Student's *t* test. Source data are provided as a Source Data file.

Supplementary Figure 3

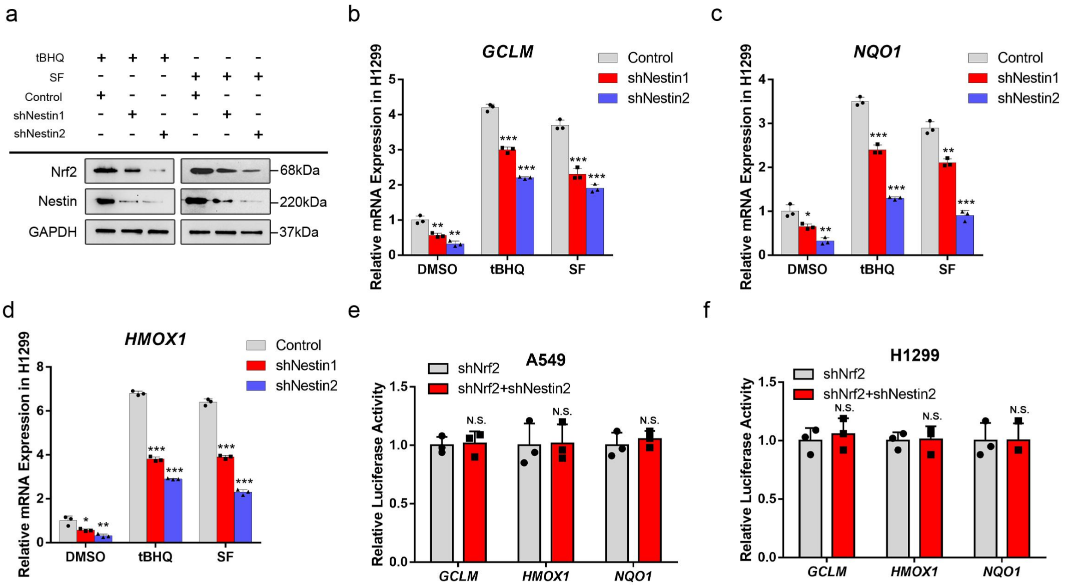

Supplementary Figure 3, relative to Figure 2. Knockdown of Nestin contributes to the deficiency of Nrf2 and its downstream ARE genes.

(a) Induced Nrf2 protein levels are lower in H1299 cells with Nestin knockdown. H1299 cells were treated with 100 μM tBHQ or 20 μM SF for 16 h. Endogenous Nrf2 was detected with an anti-Nrf2 antibody.

(b-d) The mRNA expression levels of the Nrf2-downstream genes, GCLM (b), NQO1 (c) and HMOX1 (d), were reduced in Nestin-knockdown H1299 cells. H1299 cells were treated with 100 μM tBHQ or 20 μM SF for 16 h, and qPCR was used to measure the relative amounts of the mRNAs for GCLM, NQO1 and HMOX1.

(e and f) Overexpression of Nestin is not notably affected by depletion of Nrf2 in A549 cells. Nrf2-knockdown NSCLC cells were transfected with Vector or Myc-Nestin. The results carried out via detecting the Nrf2 downstream genes expression by qPCR.

* $P < 0.05$, **$P < 0.01$ and ***$P < 0.001$, Student's *t* test. Source data are provided as a Source Data file.

Supplementary Figure 4

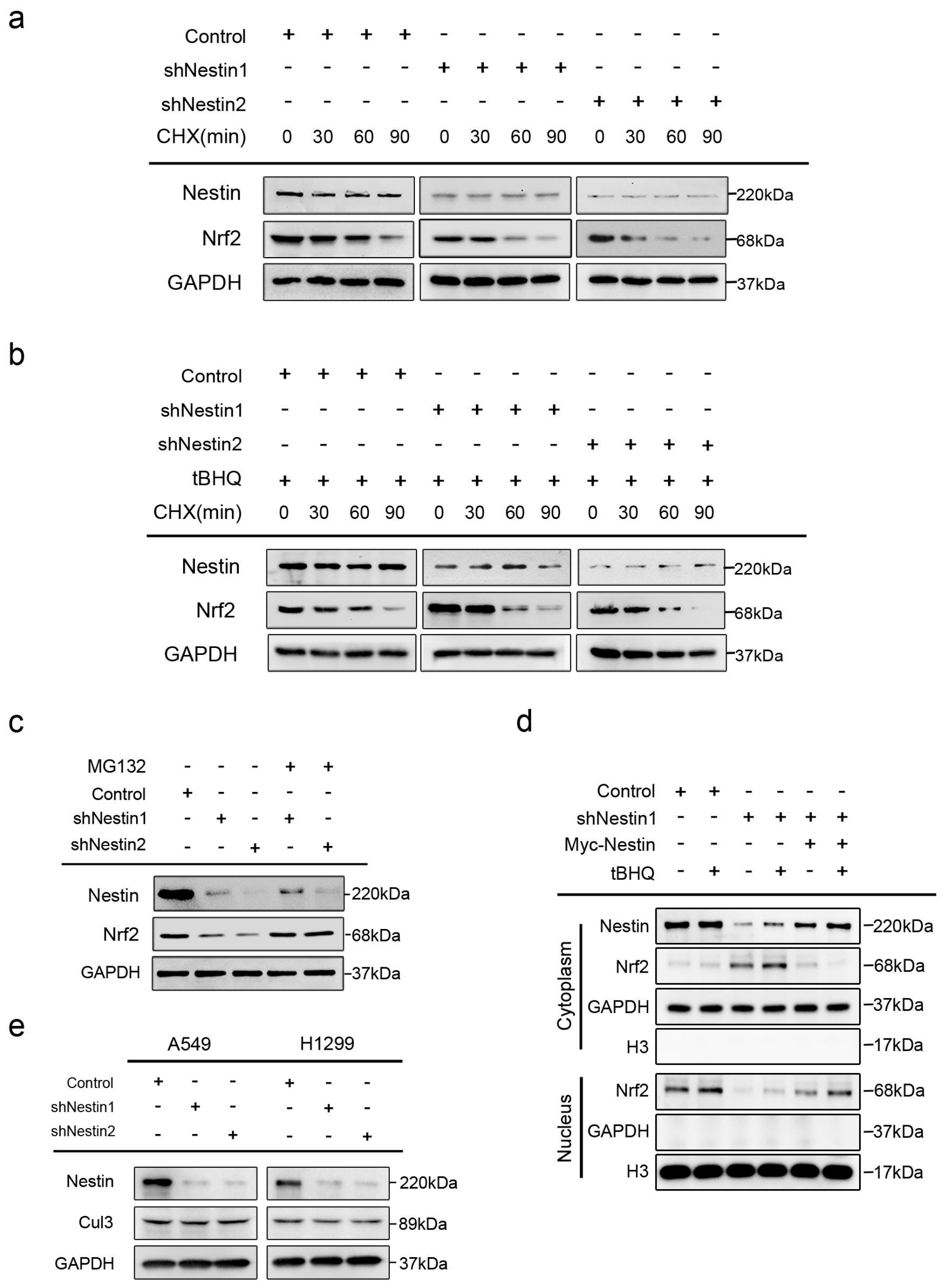

Supplementary Figure 4, relative to Figure 3. Nestin prevents Nrf2 from degradation.

(a) H1299 cells transfected with/without shNestin1 or shNestin2 were treated with 10 mg/ml cycloheximide (CHX) for the indicated durations. The protein levels of Nestin (top

panel), Nrf2 (middle panel) and GAPDH (bottom panel) were analyzed by Western blotting.

(b) H1299 cells transfected with/without shNestin1 or shNestin2 were treated with tBHQ (100 μM, 4 h) followed by 50 μg/ml cycloheximide (CHX) for the indicated durations. The protein levels of Nestin (top panel), Nrf2 (middle panel) and GAPDH (bottom panel) were analyzed by Western blotting.

(c) Western blot analysis of H1299 Nestin-knockdown and control cells treated with 10 μM of MG132 for 4 h. The lysates were analyzed by immunoblotting with the indicated antibodies.

(d) Knockdown of Nestin via treated A549 cells with shNestin1 vestors increases the nuclear translocation of Nrf2, and this effect can be rescued by overexpression of Nestin. NSCLC cells transfected with control, Nestin-knockdown (shNestin1) or Nestin-overexpression (Myc-Nestin) plasmids were treated with or without 100 μM tBHQ for 4 h. Subcellular fractionation was used to isolate cytoplasmic and nuclear proteins, and immunoblotting was performed to examine the localization of Nrf2 following the downregulation or overexpression of Nestin.

(e) Immunoblotting was performed to detect the expression of Nestin and CUL3 (cullin3) in NSCLC cells with or without Nestin knockdown.

Source data are provided as a Source Data file.

Supplementary Figure 5

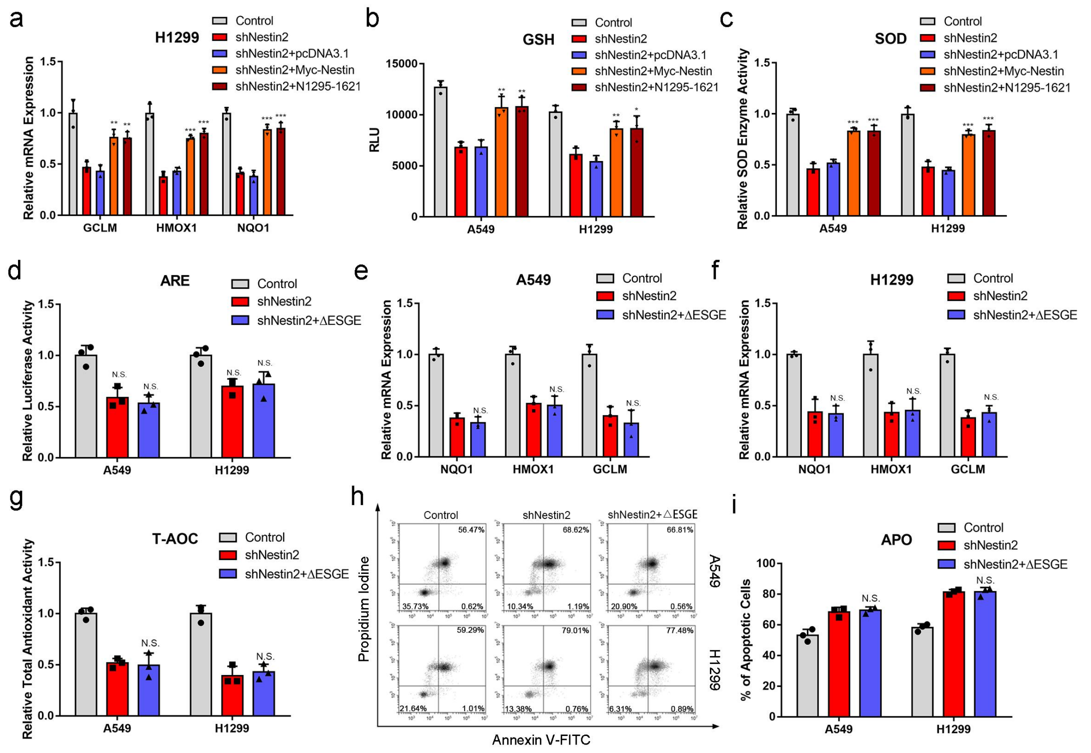

Supplementary Figure 5, relative to Figure 5. The ESGE motif plays a key role in the interaction between Nestin and Keap1.

(a) The mRNA expression levels of Nrf2-downstream genes were compared among Nestin-knockdown H1299 cells transfected with/without pcDNA3.1, Myc-Nestin or Nestin (N1295-1621) vectors. The relative mRNA levels of GCLM, HMOX1 and NQO1 were measured by qPCR.

(b) Analysis of GSH levels in NSCLC cells treated with shNestin2, shNestin2+ pcDNA3.1，shNestin2+Myc-Nestin or shNestin2+Nestin (N1295-1621).

(c) Analysis of SOD levels in NSCLC cells treated with shNestin2, shNestin2+ pcDNA3.1，shNestin2+Myc-Nestin or shNestin2+Nestin (N1295-1621).

(d) A luciferase assay was used to detect transcription driven by the antioxidant response

elements (AREs) of Nestin. NSCLC cells were treated with shNestin2 or shNestin2+Nestin (△ESGE). The results are expressed as the fold-change of luciferase activity with respect to that of the vector control.

(e) Transfection with the Nestin (△ESGE) vector failed to rescue the effect of Nestin knockdown on the expression of Nrf2-downstream antioxidant genes in A549 cells. The relative mRNA levels of GCLM, HMOX1 and NQO1 were measured by qPCR.

(f) Transfection with the Nestin (△ESGE) vector failed to rescue the effect of Nestin knockdown on the expression of Nrf2-downstream antioxidant genes in H1299 cells. The relative mRNA levels of GCLM, HMOX1 and NQO1 were measured by qPCR.

(g) The total antioxidant activity (T-AOC) was assessed in NSCLC cells transfected with shControl, shNes2 or shNestin2+Nestin (△ESGE).

(h) Flow cytometric analysis with Annexin V-FITC and propidium iodide (PI) was used to detect apoptosis and necrosis.

(i) Statistical analysis of the total apoptosis rates in NSCLC cells.

Error bars represent the SD of data obtained from three independent experiments. * $P < 0.05$, ** $P < 0.01$ and *** $P < 0.001$. N.S. represents no significant, Student's *t* test. Source data are provided as a Source Data file.

Supplementary Figure 6

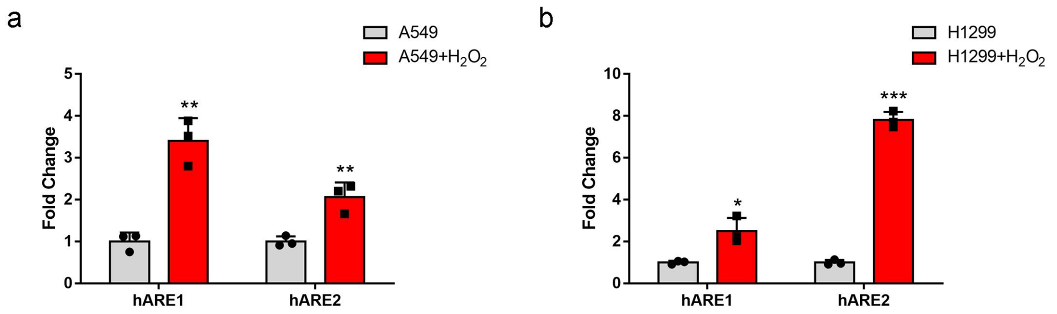

Supplementary Figure 6, relative to Figure 6. Oxidative stress increases the binding of Nrf2 to the AREs of the Nestin promoter.

(a and b) A549 and H1299 cells were treated with PBS or H_2O_2, and chromatin immunoprecipitation was performed using Nrf2-specific antibodies. DNA isolated from the precipitated materials was analyzed using qPCR with the indicated primers. The ARE-specific signals from Nrf2-precipitated DNA were normalized to those from IgG-precipitated DNA.

The data shown are means ± SD of triplicate wells. * $P < 0.05$ and ** $P < 0.01$, Student's *t* test. Source data are provided as a Source Data file.

Supplementary Figure 7

a

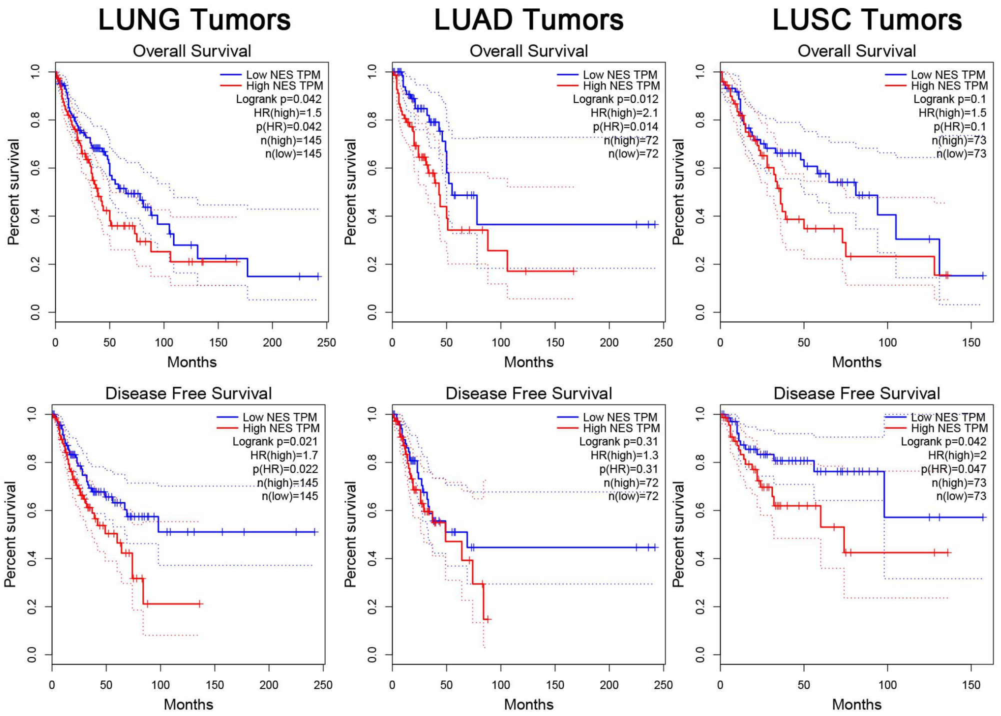

Supplementary Figure 7, relative to Figure 8. The prognostic significance of Nestin expression on lung cancer patients based on TCGA databases.

(a) Overall Survival (OS) and Disease Free Survival (DFS) in the two lung cancer patients groups of 15% cutoff high and 15% cutoff low Nestin expression, were analyzed using the GEPIA website (http://gepia.cancer-pku.cn/index.html). Kaplan-Meier OS and DFS curves of the TCGA lung cancer cohort show that patients with high Nestin level had a worse prognosis than patients with low Nestin level (OS: log-rank P=0.042, HR [high] =1.5; DFS: log-rank P=0.021, HR [high] =1.7). In detail, when Nestin was high expression, the patients with LUAD (Lung adenocarcinoma) or LUSC (Lung squamous cell carcinoma) represented significantly poor OS (log-rank P=0.012, HR [high] =2.1) or DFS rates (log-rank P=0.042, HR [high] =2), respectively.

Supplementary Figure 8

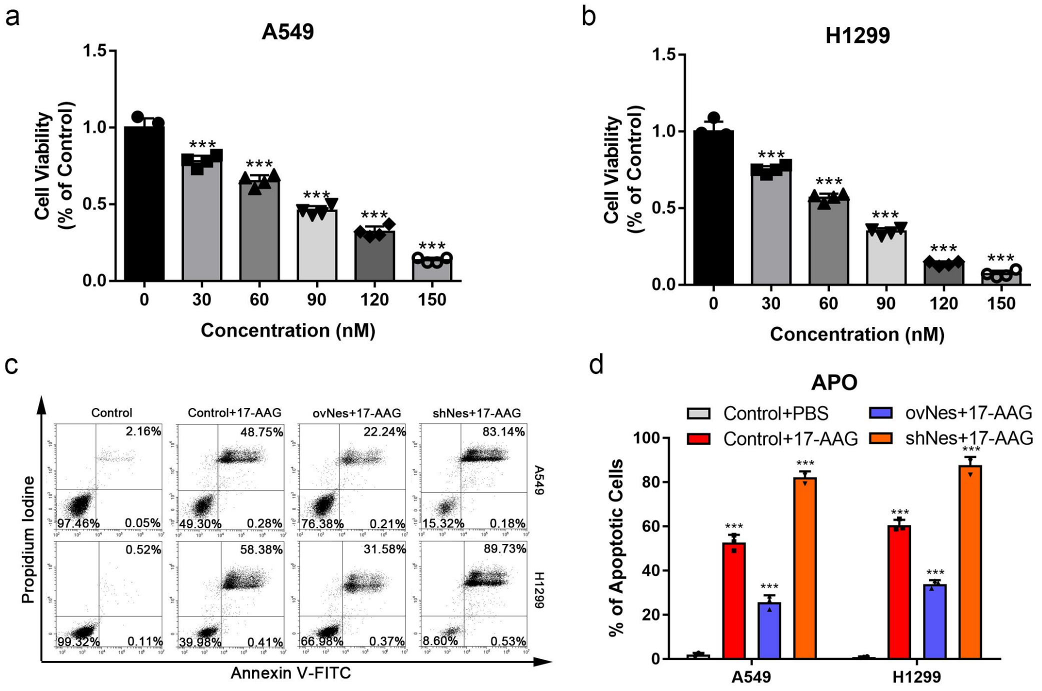

Supplementary Figure 8, relative to Figure 8. Overexpression of Nestin leads to the decline of 17-AAG antineoplastic ability.

(a-b) A549 and H1299 cells were treated with various concentrations of 17-AAG (0, 30, 60, 90, 120 and 150 nM) for 48 h. The cell viability was assessed by CellTiter-Glo assays.

(c) NSCLC cells were transfected with Control, shNestin2 or Myc-Nestin plasmids, and treated with 17-AAG according to the IC50 indicating in A and B. Flow cytometric analysis with Annexin V-FITC and PI was used to detect apoptosis.

(d) Statistical analysis of the total apoptosis rate in NSCLC cells.

Each experiment was repeated at least three times. ***P < 0.001, Student's *t* test. Source data are provided as a Source Data file.

Supplementary Table 1. The complete hARE sequence alignment in the Nestin promoter.

TGCTGAGGTGCAAGGATCATTTGAGCCCAGGAAGTGAAGCCTGCGGTTAGCTGTGATCTTGCCACTGCACT
ACAGCCTGAGAGACAAAGCAAGACCCTGTCTCAAAAATATATATATAATCTATCTATATATATTTATATATAAACA
CTACATATGTAAGTATCTATAAATAACATATTGTTTGGTTTGGCAGGGTTTTAAACGTTTAAATAAGTGGCATCC
ACTGTGCATATTTTACTGCAGCTTGCTTTTCCTGCTCACATGATGTTTGCAATTTTCATTCATTTAATATTTCCCC
AGTTGTATTAAGTGAGCATAGTTCACAATTGTGAAAAATTTAAAAGTCTCACAGTACCGCCAGGCATGGTGG
CTCATACCTGTAATCCCAGCACTTTGGGAGGCCCAGACGGGTGGATCACTTGAAGTCAGGAGTTCGAGACC
AGCCTGGTCAACATGGTGAAACCCCGTCTCTACTAAAAATACAAAAAAATTAGGTGGGCATGGAGGCATGA
GCTTGTAATCCCAGCTACTCGGGAGACTGAGGCAGGAGAATCGCTTGAACCTGGGAGGCAGAGGTTGCCA
CAAGCCAAGATCATGTTGCTGCACTGTAGCCTGGGTGACAGAACGAGACTCCATCTCAAAAAATAATAAATA
AATAAATAAATAAAAGCCTCACAGTACCAAGAGTAAATTGGTATGGGCACTTTTTTTTTTTTTTTTTTAAGAC
GGAGTCTTCCTCTGTCACCCAGGCTGGAGTACAGTGACACAATCTTGGCTCACTACAACCTCCGCCTCCTAG
GTTCAAGCCATTCTCCTGCCTCAGCCTCCTCAGTAGCTGGGATTACAGGCATGTGCCACCACGCCCAGCTAAT
TTTGTATTTTTGGTAGAGACTGGGGTTTCTCCATGTTGGTCAGGCGGGTCTTGAACTCCTGACCTCAGGTGA
TCTGACCACCTTGGCCTCCCAAAGTACTGGGATTACATGCATAAGCGATCGTGCCTGGCCACTGGTATGAGC
ATTTTTTAAAGCAATGTGGGCCGGGCACAGTGACTCACACCTGTAATCCTAGCACTTTGGGAGGCCGAGGC
GGGTGGATCACTCGAGGTCAGGATTTCGAAAGCAGCCTGGCCAACATGGTGAAACCCCATCTCTACTAAAA
ATACAAGAAAATTAGCTGGGCATCATGGCAAGTGCCTGTAATCCCAGCTACTTGGGAGGCTGAGGCAGGAG
AATTGCTTGAACTCGGGAGGTGGAAGTTGCAGTGAGCCAAGATCGAGCCACTGCACTCC

-8948 hARE5 **-8938**

AGCCTGGATGACAGAGCGAAACTCTGTCTTCAAAATAAATAAATAAATAAATACAACAACTTGGGGGCCAGG
TGCAGTGGCTCATGTCTATAATCCCGGCACTCTGGGAGGTCGAGGCAGGCGGATCACTTGAGGCCAGGAGT
TCGAGACCAGCCTGGCCAACATGGTGAAACTCCATCTCTATTAAAAATACAAAAAATTAGCCGGGTGTGGTG
GTGTGGGCCTGTAGTCCCAGCTGCTTGGGAGGCTGAGGCAGGAGAATCACTTGAACCTGAGAGGCAGAG
GTTACAGTGAGCCAAGATCGCACCACTGCACTC

-8630 hARE4 **-8620**

CAGCCTGGGTGACAGAGCAAGACTCCATCTCAAACAAACAAACACAAATCAATGTGGCATTATCAGGTGAA
GCTGAAGATATGCTTTCCCTACGACCAGCAATTCCCTCCCAGGTCGCACCTGAGAAATGGGCATGTGCATGT
GCCCAGGAGCACGGGCAGGAGCGTTCACAGCAGCACAATCAGTAAAAACCTAGACACCCCTTTATTCATTA
ATTTATTCATTTCATAAGCATACTTTGAGTACCTGTGGAGGATCAGGCACGGGGAATTTGAATAAGATAGACC
CAGTCCCTTACAGGGGTGATCGCTCTCTGCTCGGATGCGGTGGGTATTATTGTCCCTTTTTATAGATAAGGAA
GCTGAGACTCAGAAAGATTAAGTGCCTCACCAGAACTCAAACTCAGGTTTGCCTCCAGCACCTATGCTCTTT
TTTTTTTTTTAGGTGGAGTTGTGCTCTTGTTGCTCAGGCTGGAGTGCAATAGCTCGATCTCGGCTTACTGCAA
TCTCTGCCTCCCAGGTTCAAGTGATTCTCCTGCCTCGGCCTCCCAAAGTGCTGGGATTACAGGTGCCCACCA
CCACACCTGGCTAATTTTTTGTATTTTTAGTAGAGACGGGGTTTTGCCACATTGGTCTGGCTGGTCTCAAACT
CCTGACCTCAGGTGATCTGCCTGCCTCGGCCTCCCAAAATGCTGGGATTACAGGCGTGAGCCACCGCTTCCG
GCCAGCACCTGTACTCTTAATAGCTTTGCCACACTGCTTCTGGATAATGCATCTTTCACTCGTCAGCACACTGT
GGTATCTGTGCCCTCCTTCAGTGGGGTGTTCAGAGCCACACTCAGCCCTGGAGATGTGATCAGGCTAGCATG
AGGGTGGACATTTAGTCAGCTCCCTAATGGGAACTTAGTGTGGGAGGGCACAGAGGTACTGCCAAACATAG
ACCTCAGCTTCAGCCTCTCCTGGCAGCCCAGCCCAGCCTAACCCTGGCCCCCTAATCTCAGCTCCAGCCCAG
CCCCACCTGGCCTTTAGTTCTCCTCTATTTACTTCTCCCAAAAACTAGGTAGGTGGGTGGTGATGGGATAGGA

AGACTAGATTTTAACCTAGGGAGAGGGTTTTGAATCCAGAGCTGGTCATTTCCTGGCTCCAGGGGAGCTAA
GCTGGCCCTGAGTGAGATGGTAGGAGGAGCATGAGGAATGGCAAAGATACTGAAGGCTCCCTACTAGTTCC
TCTCTGATTCATCCTCTGATCCCCCAACATCGCCTTCTTTCCCAGAAAGAAGGAGGTACAGGCTGAGTCAGA
AGTGAAGTTCAAGTTCTAGCTGTGTGATCTTGGATAGGTTACTCAATTTCTCTGAGTTGTTTCCTCAGGTAGA
AAACTGGATGATAATGATGGTGATATTACCTGTCTCACTATGCAAATCTTAAGGTTTTCAAAAATTGTTTCCCA
GAGAATGTAGCTTTTCCTGAGGCAATAATGGCCCCTGGTGGCCATGCCCCAACAGAACCCACTACTTTCTGC
AGAACAGAGACCTGCGCTGAGTGAGGAAGTGGGCAGGGTGCTTTTCTGAAGTTGCTCATCAAAACCACAG
ACCCAGGTTGGGAGTGGTGGCTCATGTCTGTAATCCCAGCACTTTTGGGAGGTCGAGGCAGGCGGATCACC
TGAGGTCAGGAGTTCGAGACCAGTCTGACCAATATGGTGAAACCCTGCCTCTACTAAAAATACAAAAATTAG
CCAGGCATGGTGGTACGTGCCTGTAATCCCAGCTACTCGGGAGGCTGAGGCAGGAGAATTGTTTGAACCCG
GGAGGCAGAGGTTGCAGTGAGCCAAGATTGCACCACTGCACTCCAGCCTGGGTGACAGAGTGAGACTCTG
TCTCAAAACAAAAACAAAAACAAAAACAAAACAAACAAAAAACCCCACAGACCCAGAGAGTTAGGGTTAA
AAGGGAACAGCTTGTTGTTTTATACCCTTACCCTTATCCTCTGCGACCTCATCCATGCTCTTAGTCGCCCTTTAT
CCACTCTAACCAGGCAGCATCCTTCTAAATCTCTATCCCAGCCCCCAATCCTAAAACTGCATATCTGGAAGCTG
CTGTATCTCCCCCATGCCACTATCTCTCCCCTTCCCCAAGGCTTAACCACATACCTGGGAGTCTTCCCTGTCCC
CTCCCTCCAAACCCACATCTAACTCATCCTGGAACACACACAGCCTAGAAATTCTCATGTCTCTTCCCTCCTCT
CCACCCCTAGGCTCAGGTCTCATCCAACCTTGCTAGTGGCAACTGCCTTCTAACTCGCCACTACCCCAACCCT
CCAAGATGATCTTTCTAAGATGCAAATCTGACCAGGCTACCCTTTGGTTGAGGTTAAAACCCTTCATGGCTCC
TCGCTGCCTAGAGGACAAAGGCCGATGCTCCTCCCTTCACCATGCCTGCCCCCTCCTCTAACTTCCCTTGCTC
ATGCTTGCCTGTCTGCACGGGACATACTCCCTGGCTCCCAGGCCTGCCCTCTAACACACCCATAGGCTCCTTG
GGGAAGGGCTCCATATCGGTCACCTTTGATTCCTTTGCAACATTTATTGGGTGCCTGCCATGCACCTGGTATT
CAATAGGTGCTTAATAAATATTGTTTGGCCAGGTGAGGTGGCTCACACCTATAATCCTAGCACTTTGGGAGGC
CGAGGTGGGTGGATCACTTGAGCCCAGACGTTCAAGACCAGCCTGGGCAACACAGGAAAACCCCATCTCT
ACAAAAAATAAACAAAATTAGCTGGGCATAGTGGCATGCACCTGTGGTCCCAGCTACTCGGGAGGCTGAGG
TGGGAGGATCACTTGAGCTCTGGAGGTCGAGGCTGCAGTGAGCCCTGATAGCACCACTACACTCCAGCCTG
AGTGACAGAGTGAGGCCCTGTCTCAAAAAGAAAAAAAGCTGGGCTCGATGGCTCACACCTGTAATCCTAGC
ACTTTGGGAGGCCCAGGTGGGAGGATTGCTGGAGCTCAGGAGATTGAGACCAGCCTGGGCAACACAGTG
AGACCTCTTCTCTACAAAAATAAAAAATAAAAAATTAGCTGGGTGTGGTGGCACGTGCCTGTAGTCCCAGCT
ACTTGGGATGTTGAAGCAGGAGGATCGCTTGAGCCTGGGAGGTTAAGGCTGCAGTGAGCCATGACTGCA

-5450 hARE3 **-5440**

CTATTGCACTCTAGCCTGGGTGACAGAGCGAGACCCTGTCTCAAAAAATAAAAGGAAGTAGAATTTAAAAA
AATAAATCAGTAAGGAAACCAAGTCCCAGAGGGGTGACTTGATTATTTCAAATATCCCATAGTGACACAGGG
CTCCCCGTTCAATGTTTTTTTCACTCCACTGGGCTGCTCCTTAGCTTCTACCCCCAGGACAGAGAATGGGAA
GGCCACCTTTCCTGCTGTGGACATGAAGGGAAGGAGAGGGCAGGAAAGCCAGTCCCATCATCCCTCCAGC
AGGGAAAGGAGAAAATAGAGTGAGAAGGGACATTCCCACCTACTGGTGCCAGCTCCAAGCCTCCCTCCTGT
GGCCGGGGAAAAGCAGGACAGAAAGGCAGGGCCCAGCCAGGATGCAGAGCTCCCAGGTCCCAGCCAGG
CTGCCCAGAAGAGGCCCTTCCCTTTCCCTGCTTCCCAGGTGCCTTGGTACAGAGCTTCTCAAAGTTGCTCCC
CTGCCACCTGGTTTCCTTCTCCTGTTTGTTTCCTTCTCCCCATCTCTTTTCTAAGACTCATTGCTGACCTTATCC
TTGCCACATGCGTCCAGTAAGACCTGTCTAGCTGGGAAGTGGAGGGAAAGGCAGAGTAGAGGAGGGGCT
AAGTTAGGGATGAGGGCAAGAGCGACTTCCTGGCCCCTGCCCTAGCCCATGCATTGTGAGGCAAAGAAGG
TAAGGGATAGGGTAGGTAACAAAAATGAAAGAGTTTTAGATCTGTCCGACCACCGCCTGCAGTGTTCTGTTG
TATCTCCTCCTCCTGTCTTATAATGACTTTTTGGCAGATTCTAGAGCTTTCTAGGTACTTTGGGCTATACAAGTC
CTATATAGTTCAGTTGACCCAGTTTTAAACTTCAATCTCCTGTCCCATTAAAACACTCTTCATTACCTGCTAGCC
AAGGCTTGGCCCCAGGTCCAAAGGCTGGCACTGGGGAGAGGAATGCTCTGCTCTTTGCACCCACCCCCAC

CCCTGTGGCCCCCTCTGAGGCTTATTTCCCCGCTGTAGGAGAGAGGAAGACACAGGGGTAGGTGCCACTTA
CCTAGTGTTACAGTCAACCTCCCTCAGACGTGAAGCTCCCTTGGGTGTGGTGACACCCAAGCCGGCTGCAC
CATGGGGGCTCCGTTTGTGGGGTCCTCTTGGCCCACAGAATCTCACCTGTCTCCTCCTGTGCCCCTCTCCTTT
CCCCATTCATCTCTCTCCAGGCTCTGTGTCCCTTATTCAGTGAGGCTAAGAGAGTGGATGAGCAAGTCCTCCA
CCCTAAAAGACAGCCGGCCTGGAGCGGGATGCCAATGTGTCCTTTGGCAGGCCCTGCCTTGAGGAGTCCTT
CCTTGGGGAGGCTGCACCCACTCTCCTTGGTGGGCATCATCCTTGCCCTCTGAGCAGAGGAGGGAAAGCCA
CAGCCTGCCATCCTGGACTGGCCACTCCACACTCCAGTGCTGCTGGTCACAGTCTCCTCACATTCCGCTTGCC
TTCTCTAATGCAGACTTCGGTGGGGAGAAGGCCTTCAGGGTGGGGGCTGGCAATTTCCTTTAGGCTGTGTG
TGTGCTACTCAGAGGCCATTATTTTTAGTTTCAGAGCTTAGACACAAGTTCATTCAGAAAGTCTTTATCAAAC
ACTCTGGGACGGGTGTGGTGGCTCACGCCTGTAATCCCAGCACTTTGGGAGGCTGAGGCAGGTAGATCACT
TGAGGCCAGGAGTTCGAGATCAGCCTGGCGAACATGGTGAAACCCCGTCTCTACTAAAAAAAACACAAAA
AATTAGCTGGGCATGGTGGCACAAGCCTGTAGTGCCAGCTACTCGGGAGACTGAGAATCACTTGAACCGGG

-3557 hARE2 **-3547**

GAGGCAGAGGTTGCAGTAAGCCGAGATCACGCCACTACACTCCAGCCCAGGTGATGGAGCGAGACTGTCT
CCAAAATAAATAAATAAAATTTTAAAAAATTTAAAAATTAAAAATACAAAAACATTATTCCAAGATCCCTTACC
ATCTGGTCATCTTCTTCTGGGATTAACTCCAATCCATCAAGGTCCCTTTTAAAATATAGTGTCCAGTACAACAC
TCCAGATGTGGAGGGCAATGGGAGTGTTGCCATTGCTCATCTGGACACCTTGAATCTATAGTCACTTTTATCC
TTTTCACTTTTTTAGACAGCCACCCAGTATTGGTTTCTTCCTAACAAGCTTGTAGTCTTCTAAAACCCCCAGCT
CTTTCTTCTTTGCTAGACACTCCCTACTCTTCAGCCGGTCTTTATTCTCCTCTTCTTATGCTGTTGATTTTTTGA
AACCAAAAGCAGAATTTTACACTTATCCCTATTAAATGTCATCTGTTTTTTTTTTCTATTTTGCTAAAATATAACT
CTCAGTTCTTGCATAGTCTGTAGATCTGATAAACATGGCTTCCATGTTTTCATTCAAGTCTTTAATTAAAACTTT
TAATTGTCAGGACCAAGGACAGGTTCCCTCGGGGCAGCACACAGAACCCACTAAGCTTGCTCATGGCCATC
TCTGGGGATGACTGCTGGGCTGACTTTGTATCCACCAGCTGTAGTACCACATAGTTCATATGTCCTGGGTCCA
GAGACCCAATAAAAGGTACGGCCAATGGCCCCACAAACATCCCAGTAACTAAATGCAGTCACCAGGGAAGG
AGATGAAGGGAGGGCCTAAGTTTCTCTAGGGAGGGCCTTATTTCTCTAAGTACCTCAAATATCAGGTATCTGT
TTAGAATCTGGTAAGCACTGACGTCAGATCCTCTAGGTTGTGAATGCTGGAACCTACCATCTCCTGTTTTAGG
AAAATCAACACCACACTCTTCCCCAGAATTCTTAACAATCTCTATTCCCCATAATTCCTAAAACTCACAAGAGT
GATTCACAAGAGGAGGTTTTCTTTAGCTTAATGGCTTTCATTGTGACGGTCCTCAAAAAAGGCTTTATACAA
AGACTTCCCTATACCTGGTCTTATTTGATTATCAGAACAAACTTATAGGGATAGGTGGGTATTAACCTGCTTTG
CCAAATAGGTAAACTCAGGCTCACTTAGAGAAGTTATGTGATTTGTCCAATGTCATTCAGTTAGAAAGTGCAA
AAGTCAGCTAATCTTTGCATGAGACACAGTTCCAAATGTCTCCCCTTTGGGTGAGGGTGAGGGGTTCAGAG
AGGAACCCCCAGCCCAATGCCACCCTACCTCTTTAGGGGACCGCCCCCAAGAATCCCAGGGGATCTCAAAC
ATTTAGCATCCCTCTCCTCCCAGACAGAAAGTGAAAGAAAGAAGGAGGTCGGGTGCAGTGGCTCATGCCTG
TAATCCCAGCACTTTAGGAGGCCAAGGCGGGTGGATCACCTGAAGTCAGGAGTTCAAACCAGCCTGGCCA
ACATGGTGAAACCCCATCTCTACTAAAAATACAAAAATTAGCCTGGCATGGTGGCAGGCATCTGAAATCCCA
GCTACTTGGGAGGCTGAGGCAGGAGAATCACTTGAACCGGGGAGGCGAAGGTTGCAGTGAGCCGAGATC
ACATCACTGCACTCCAGCCTGGGCAACAGAGCAAGACTCCCTCTCAAAGATAAAAATAAAAATAAAAGAAA
GAGAGGAGGAGATTCCAAGCAAGCTCTGCTGGCCACAACTCTGGGGAGCCCTCAGCCCAAACCAAACAGC
AGGATTTATGAGGTAATGCAAGGTTGAAGGAGCCTGGGCATCCCAGATCCTCAGCCTCCCGGCCTGCACTG
ACTCTCAGTCAATGTCCTGTCACTAACCCCTCCCTGGCCTCTATTCTACATATTGGTGGTGAATTCCAAAGCCC
CATTGGCCTGGGGACAGGGATGGGGTCCTTGAGAGACTCCTCAAGGGGAGGGGATATTGGAGGGATGAC

-1573 hARE1 **-1563**

TTCCTGGTTATTGACCCGGCTGTACCCTCTACTCAGAAAACCCTCTCCATCCTCTGTGCTGCCTGGCTCTGTCC
CCAGCACCTTGGAGGCTGATTAAGTATTGTAGTCATCTTTTTATGTTTACCTCCACCAACAGACTATGAGTGCC

CATCACTGTTGACCTCACATCACCAAACTATAAGCATTTCCAGGGCTGGGACAGAGTTCCTGCAATCTCTTTG
GTGCTGACACATAGTAGGTACTCAATTAACACTTGAGCTAAATTGGATTTAGCAGCTTGATCAGAAACGCCTT
CCCCAAAGATCCCCTCCGTCTCATTATCCCTGCATCTCCTCCCCAGCCCTACTTCTCCTTCACTGTAAATCCCCC
AAATCCCACCTGTCCTTTACCTCCCAGCTCAAGGATGCACTCCGTCCAGAAAGCCTTCCCTACTACTCTCACC
CTTGGTGAGCTCTCTACTTCATAAACTGTAGAATCTTTAAAAGGTTGTTTTGAAGATTAAGTCAGATCATGGA
AGTAAAATATTGGAACACGACTGGGCACATCATAAAGGCTGGATAAACTTGGGCTAGTCGTTAGGAAGGAA
AAGAATGGCCTCTTCCTGCTGCTTCCACTTCTGAAAGCCGCCAGAGGGCGTCCACTACCCAACATTCCCTGG
CAGGGGGTGCGGGAGAAGGGGCCCTTCCCCAAGAACAGAACTTCCTAAAGCGGATGTTTGAACCTCGCAG
TTATACAGAAGACTTGTAGGAAGGATGGACAAACGTTCTTAAGCCCATGACGGCCCTTAACCTGGTCGCTCC
CTTTTCTGATGGAGACTCAGGCAATAGCGTGTGTGCGTGTGTGTGTGTGTGTGTGTGTGTGTGTATCCGT
GTGTCCTAATATCAGACATTTGTTCTTGTTTTCCAGGCAGCGTCTCTCTAGCTTCTTTCTGCAATGCTGTAGTA
CTCTCTCCAGTATTTCAGGAGGAGGAGCATTTGCTATTTCAAAAACGAAAAACAAAAACCTGGCCACATCCA
TTTTTTTCAGCAGCCATGCGATTTCCATCATTGCTCACATTTTATGGATGAGGAAACTGAGTCTTAGAGGAAT
TCAGTAAGTGATACCTCTCTCGGATGTGTTGAGTAACTGAGACTGCACTCCCTCCCAGGCTGGAACGTCCTG
GTACTCCCACCCCCACAGGCTCAGTTCTGTGCATTATCTGCCTTTTTCGGGGATTGTGACCCTTCTTCACAGC
CTCCTCCCTCAGAAAGCCACCACCATCAGATCCGATTCTCCATGGTACAGCTTCTTCTTTGGTTCCACTCTCCA
GCACCCTGGGGAAGCAGGAACAGAGGCTGCTGCCACTCTCTGACCTCTAAGGGGTTAAGGCCTGGGTCCC
GCCCCTCTTCCCGCCCGCCTGGCGGGAGTATGAATAGCCTCGCTCCCACTCCCGACTCTCAGTCGCTCAGGC
TACTCCCACCCCGCCCCGCCCCGTCATTGTCCCCGTCGGTCTCTTTTCTCTTCCGTCCTAAAAGCTCTGCGAGC

-1 | +1

CGCTCCCTTCTCCCGGTGCCCCGCGTCTGTCCATCCTCAGTGGGTCAGACGAGCAGG|ATG

Supplementary Table 2. Target sequences of shRNAs. Related to Experimental Procedures.

Name	Sequences (5′ to 3′)
NESTIN shRNA#1	5′-GCTAGTCCCTGCCTGAATA-3′
NESTIN shRNA#2	5′-GCAGACATCATTGGTGTTAAT-3′
NFE2L2 shRNA	5′-CCGGCATTTCACTAAACACAA-3′
NESTIN siRNA	5′-GCAGACATCATTGGTGTTAAT-3′
NFE2L2 siRNA	5′-CCGGCATTTCACTAAACACAA-3′

Supplementary Table 3. Plasmids used related to Experimental Procedures.

**Plasmids:**	**SOURCE**
POZ-N-FH-Flag-Keap1	Dr. B. Xia
POZ-N-FH- Flag-Keap1 NBTB	Dr. B. Xia
POZ-N-FH- Flag-Keap1△KC	Dr. B. Xia
POZ-N-FH- Flag-Keap1 KC	Dr. B. Xia
POZ-N-FH- Flag-Keap1△C	Dr. B. Xia
pcDNA3.1-Myc- vector	Invitrogen
pcDNA3.1-Myc-(1-1621)-Nestin	This study
pcDNA3.1-Myc-(8-313)-Nestin	This study
pcDNA3.1-Myc-(314-640)-Nestin	This study
pcDNA3.1-Myc-(641-1621)- Nestin	This study
pcDNA3.1-Myc-(1295-1621)- Nestin	This study
pcDNA3.1-Myc-ESGE1417mutant Nestin	This study
pcDNA3.1-Myc-△ESGE Nestin	This study
pTRE3G-IRES	Clontech
pLVXTet3G	Clontech
pLVXTet3G- EF1α	This study
pTRE3G-IRES- shNestin	This study
pLV-Flag-Nrf2	This study
pLV-Myc-Nestin	This study
pGL3-basic luciferase reporter plasmid	Professor Jun Li
pGL3-basic luciferase -NestinARE1	This study
pGL3-basic luciferase -NestinARE2	This study
PHBLV-ZsGreen-fLUC	Hanheng Biotechnology

Supplementary Table 4. Primary and secondary antibodies.

Product	Catalogue Number	Dilution	Supplier
Primary antibody:			
WB:			
mouse anti-Nestin	611658	1:1000	BD Biosciences
mouse anti-Ub	sc-8017	1:500	Santa Cruz
rabbit anti-GAPDH	2118s	1:1000	Cell Signaling Technology
rabbit anti-Flag-Tag	SAB1306078	1:1000	Sigma
rabbit anti-cMyc	2278	1:1000	Cell Signaling Technology
rabbit anti-Keap1	8047	1:1000	Cell Signaling Technology
mouse anti-Nrf2	ab89443	1:500	Abcam
rabbit anti -GCLM	14241-1-AP	1:1000	Proteintech
rabbit anti-NQO1	ab34173	1:1000	Abcam
rabbit anti-HO-1	5853	1:1000	Cell Signaling Technology
rabbit anti-H3	ab1791	1:1000	Abcam
IP:			
mouse anti-DYKDDDDK（Flag Tag）	8146	1:50	Cell Signaling Technology
mouse anti-cMyc	2276s	1:1000	Cell Signaling Technology
goat anti-Keap1	sc-15246	1:30	Santa Cruz
rabbit anti-Nrf2	ab62352	1:20	Abcam
ICC:			
rabbit anti-Nestin	ABD69	1:500	Millipore
mouse anti-Nestin	MAB5326	1:200	Millipore
rabbit anti-Nrf2	ab62352	1:200	Abcam
rabbit anti-Keap1	8047	1:400	Cell Signaling Technology
rabbit anti-NQO1	ab34173	1:200	Abcam
mouse anti-HO-1	ab13248	1:200	Abcam
rabbit anti-GCLM	14241-1-AP	1:100	Proteintech
ChIP:			
mouse anti-Nrf2	ab246229	Use 2.0 μg for 25.0 μg of chromatin	Abcam
IHC:			
rabbit anti-Nestin	ABD69	1:300	Millipore
mouse anti-Nrf2	89443	1:300	Abcam

Secondary antibody:			
WB:			
anti-mouse IgG HRP-linked Ab	7076	1:1000	Cell Signaling Technology
anti-rabbit IgG HRP-linked Ab	7074	1:1000	Cell Signaling Technology
anti-goat IgG HRP-linked Ab	Ab6885	1:1000	Abcam
ICC:			
goat anti-mouse IgG Alexa 488	A11001	1:500	Invitrogen
goat anti-rabbit IgG Alexa 488	A11008	1:500	Invitrogen
goat anti-rabbit IgG Alexa 555	A21428	1:500	Invitrogen
goat anti-mouse IgG Alexa 555	A21422	1:500	Invitrogen
donkey anti-goat IgG Alexa 594	A11058	1:500	Invitrogen

Supplementary Table 5. Primer used to amplify the human transcripts or genome DNA during PCR. Related to Experimental Procedures.

Gene(huaman)	Sequences (5′ to 3′)	application
hNESTIN	Forward: 5'- CTGCTACCCTTGAGACACCTG-3'<br>Reverse: 5'- GGGCTCTGATCTCTGCATCTAC-3'	qPCR
hNFE2L2(hNRF2)	Forward: 5'- ACACGGTCCACAGCTCATC-3'<br>Reverse: 5'- TGTCAATCAAATCCATGTCCTG-3'	qPCR
hKEAP1	Forward: 5'-ATTGGCTGTGTGGAGTTGC-3'<br>Reverse: 5'-CAGGTTGAAGAACTCCTCTTGC-3'	qPCR
hGAPDH	Forward: 5'- CTGACTTCAACAGCGACACC-3'<br>Reverse: 5'- TGCTGTAGCCAAATTCGTTGT-3'	qPCR
hGCLM	Forward: 5'-TGTCTTGGAATGCACTGTATCTC-3'<br>Reverse: 5'-CCCAGTAAGGCTGTAAATGCTC-3'	qPCR
hGCLC	Forward:5'-AGAGAAGGGGGAAAGGACAAAC-3'<br>Reverse:5'-AAGTTATTGTGCAAAGAGCCTGAT-3'	qPCR
hHMOX1	Forward: 5′-CTCAAACCTCCAAAAGCC-3′<br>Reverse: 5′-TCAAAAACCACCCCAACCC-3′	qPCR
hNQO1	Forward: 5'- ATGTATGACAAAGGACCCTTCC-3'<br>Reverse: 5'- TCCCTTGCAGAGAGTACATGG-3'	qPCR
hHO-1	Forward: 5'-AACTTTCAGAAGGGCCAGGT-3'<br>Reverse: 5'-CTGGGCTCTCCTTGTTGC-3'	qPCR
hCAT	Forward: 5' -ACTTTGAGGTCACACATGACATT-3'<br>Reverse: 5' -CTGAACCCGATTCTCCAGCA-3'	qPCR
hGPX1	Forward: 5' -TGCAACCAGTTTGGGCATCA-3'<br>Reverse: 5'-ACCGTTCACCTCGCACTTC-3'	qPCR
hGPX4	Forward: 5′-CAGTGAGGCAAGACCGAAGT-3′<br>Reverse: 5′-CTGCTTCCCGAAGTGGTTAC-3′	qPCR
hSOD1	Forward: 5'-AGGGCATCATCAATTTCGAGC-3'<br>Reverse: 5'-GCCCACCGTGTTTTCTGGA-3'	qPCR
hSOD2	Forward: 5'-AACCTCAGCCCTAACGGTG-3'<br>Reverse: 5'-AGCAGCAATTTGTAAGTGTCCC-3'	qPCR
hARE1-NESTIN	Forward: 5'-ATCATGGCAAGTGCCTGTAATCC-3'<br>Reverse: 5'-TAGACATGAGCCACTGCACCTG-3'	ChIP-qPCR
hARE2-NESTIN	Forward: 5'-GTCCCAGCTGCTTGGGAGG-3'<br>Reverse: 5'-CTGGGAGGGAATTGCTGGTCG-3'	ChIP-qPCR
